# Protective Effects of Vildagliptin against Pioglitazone-Induced Bone Loss in Type 2 Diabetic Rats

**DOI:** 10.1371/journal.pone.0168569

**Published:** 2016-12-20

**Authors:** Young Sil Eom, A-Ryeong Gwon, Kyung Min Kwak, Ju-Young Kim, Seung Hee Yu, Sihoon Lee, Yeun Sun Kim, Ie Byung Park, Kwang-Won Kim, Kiyoung Lee, Byung-Joon Kim

**Affiliations:** 1 Department of Internal Medicine, Gachon University School of Medicine, Incheon, South Korea; 2 Department of Internal Medicine, Gachon University Gil Medical Center, Incheon, South Korea; 3 Imaging Science based Lung and Bone Disease Research Center, Wonkang University, Iksan, Jeonbuk, South Korea; Katholieke Universiteit Leuven Rega Institute for Medical Research, BELGIUM

## Abstract

Long-term use of thiazolidinediones (TZDs) is associated with bone loss and an increased risk of fracture in patients with type 2 diabetes (T2DM). Incretin-based drugs (glucagon-like peptide-1 (GLP-1) agonists and dipeptidylpeptidase-4 (DPP-4) inhibitors) have several benefits in many systems in addition to glycemic control. In a previous study, we reported that exendin-4 might increase bone mineral density (BMD) by decreasing the expression of SOST/sclerostin in osteocytes in a T2DM animal model. In this study, we investigated the effects of a DPP-4 inhibitor on TZD-induced bone loss in a T2DM animal model. We randomly divided 12-week-old male Zucker Diabetic Fatty (ZDF) rats into four groups; control, vildagliptin, pioglitazone, and vildagliptin and pioglitazone combination. Animals in each group received the respective treatments for 5 weeks. We performed an intraperitoneal glucose tolerance test (IPGTT) before and after treatment. BMD and the trabecular micro-architecture were measured by DEXA and micro CT, respectively, at the end of the treatment. The circulating levels of active GLP-1, bone turnover markers, and sclerostin were assayed. Vildagliptin treatment significantly increased BMD and trabecular bone volume. The combination therapy restored BMD, trabecular bone volume, and trabecular bone thickness that were decreased by pioglitazone. The levels of the bone formation marker, osteocalcin, decreased and that of the bone resorption marker, tartrate-resistant acid phosphatase (TRAP) 5b increased in the pioglitazone group. These biomarkers were ameliorated and the pioglitazone-induced increase in sclerostin level was lowered to control values by the addition of vildagliptin. In conclusion, our results indicate that orally administered vildagliptin demonstrated a protective effect on pioglitazone-induced bone loss in a type 2 diabetic rat model.

## Introduction

Type 1 diabetes (T1DM) is well known to be associated with low bone mineral density (BMD) and a high risk of fracture due to osteoblastic dysfunction. And regardless of the BMD status, patients with type 2 diabetes (T2DM) have a high risk of fracture, especially at the hip [1–3].

Thiazolidinediones (TZDs) are peroxisome proliferator-activated receptor-r (PPAR-r) agonists and insulin sensitizers that are used for the treatment of T2DM. Long-term use of TZDs is associated with bone loss and an increased risk of fracture in women with T2DM [4]. Post-hoc analyses of large randomized controlled trials (RCTs) have shown an increased risk of fractures with rosiglitazone treatment relative to metformin or glyburide in women with T2DM [5,6]. In addition, according to the recently reported ACCORD bone study, use of TZDs increases non-spine fracture in women with T2DM [6]. These results are related to decreased bone formation and increased bone resorption associated with the use of TZDs [7].

Recently, incretin hormone-based therapies, including glucagon-like-peptide-1 receptor agonists (GLP-1RA) and dipeptidylpeptidase-4 inhibitors (DPP4i) have been used as new treatment options to control glucose levels in patients with T2DM. These drugs have benefits in many systems including the skeletal system beyond glycemic control [8].

GLP-1RA increases bone formation and decreases the bone resorption rate. GLP-1 receptors are present in murine osteocyte cells [9]. In a previous study, we reported that exendin-4 might increase BMD in type 2 diabetic rats potentially by downregulating sclerostin in osteocytes [10]. Effects of DPP4i on the bone have also been reported. A recent study showed that sitagliptin treatment for 12 weeks attenuates bone loss and improves mechanical bone strength in streptozotocin-induced diabetic rats without any effects on glucose levels [11]. However, in humans, the effects of incretin hormone-based therapies on the bone are still unclear.

In the clinic, DPP4i and TZD are the best combination for the control of diabetes. A combination of two drugs may cover the several causes of hyperglycemia, like insulin secretion defect, glucagon over secretion, and insulin resistance. Additionally, incretin-based treatments such as DPP4i may play a protective role against TZD-induced bone disorders. The objective of this study was to investigate the protective role of DPP4i (vildagliptin) on bone mass, BMD, and bone turnover markers in TZD (pioglitazone)-treated Zucker rats.

## Materials and Methods

### Animals and treatments

Twelve-week-old male Zucker Diabetic Fatty (ZDF) rats were supplied by Orient Bio Inc. (Gyeonggi, Korea). Animals were maintained in animal facilities at the Lee Gil Ya Cancer and Diabetes Institute, Gachon University of Medicine and Science, under constant temperature (22–24°C) and humidity (40–60%) with a 12-h light and 12-h dark photoperiod. All animal experiments were conducted in accordance with the protocol approved by the Institutional Animal Care and Use Committee at Lee Gil Ya Cancer and Diabetes Institute, Gachon University (LCDI 2013–0073). After a 1-week adaptation period, the Zucker rats were divided into four groups: control (vehicle, n = 6), vildagliptin (10 mg/kg/day, n = 6), pioglitazone (30 mg/kg/day, n = 6), and combination group (vildagliptin 10 mg/kg/day and pioglitazone 30 mg/kg/day, n = 6). Each drug was administered by gastric gavage daily for a period of 35 days. Animals were checked daily for any signs of sickness during the experimental period. There were no animals that became severely ill or died at any time prior to the experimental endpoint. Animals were kille by CO_2_ exposure after 5 weeks of treatment and we observated that the animal fail to recover within 10 minutes after CO_2_ exposure ends. All efforts were made to minimize suffering.

### Measurements of body weights and blood glucose levels

The body weights and blood glucose levels were measured daily during the course of the study using an OHAUS weighing balance (Corporation, Shanghai, china), and a glucometer (OneTouch Ultra, Lifesan, Johnson & Johnson, Milpitas, USA), respectively.

### Intraperitoneal glucose tolerance test (IPGTT)

Animals were fasted overnight and glucose (2 g/kg, JW Pharmaceutical, Seocho, Korea) was administered intraperitoneally before and after a 5-week treatment. Blood samples were obtained from the rat tail vein at 0, 15, 30, 60, and 120 min glucose load and glucose levels were measured using a glucometer (OneTouch Ultra, Lifesan, Johnson & Johnson, Milpitas, USA). The area under the curve (AUC) for glucose was calculated over the 2-h period of IPGTT. Serum insulin levels were measured at 0 and 15 min using a commercial ELISA kit (ALPCO Diagonostics, Windhan, NH, USA).

### Dual-energy X-ray absorptiometry (DEXA)

Whole body BMD was evaluated by DEXA. DEXA was used to measure area (cm^2^) and BMD (g/cm^2^) using a pDEXA Sabre X-rat Bone Densitometer (Norland Stratec, Pforzheim, Germany). Quality assurance was performed by scans of the two solid-state phantoms provided with the scanner.

### Micro-computed tomography (micro-CT) analysis

The femur metaphysic regions were scanned using a high-resolution micro-CT (NFR-Polaris-S160; Nanofocus Ray, Iksan, Korea) with a source voltage of 55 kVp, current of 100 μA, and 7 mm isotropic resolution. Femur scans were performed 2 mm proximal to the growth plate, with a total of 720 sections per scan. After 3D reconstruction, bone volume per tissue volume (BV/TV, %), trabecular number (Tb.N, 1/mm), trabecular thickness (Tb.Th, μm), and trabecular separation (Tb.Sp, μm) were calculated using INFINITT-Xelis software (INFINITT Healthcare, Seoul, Korea).

### Assay of serum active GLP-1, bone turnover markers, and sclerostin

At the end of the experiment, blood was collected from animals that were fasted overnight. Serum was analyzed for assessment of active GLP-1, bone turnover markers (osteocalcin and tartrate-resistant acid phosphatase (TRAP) 5b), and sclerostin by ELISA (IBL, Osaka, Japan, osteocalcin, TRAP 5b and sclerostin; Houston, Tx, USA).

### Statistical analyses

All data are presented as mean ± standard deviation (SD). All statistical analyses were performed with GraphPad Prism 5.0 (GraphPad Software, Inc., San Diego, CA). Statistical differences were analyzed using one-way analysis of variance (ANOVA) and Tukey’s multiple-comparison post hoctest. A *p* value of < 0.05 was considered statistically significant.

## Results

### Changes in blood glucose levels and body weights during treatment in ZDF rats

**Fig 1:**
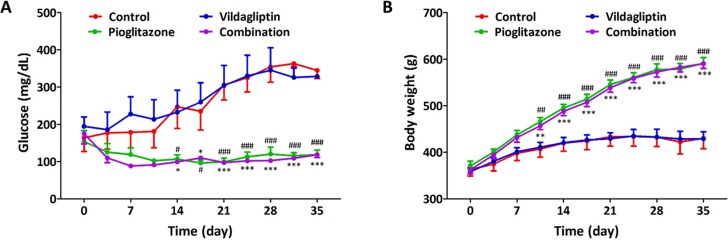
Changes in blood glucose level and body weight during the treatment in ZDF rats. (A) Change in blood glucose level during the treatment with control, vildagliptin (10 mg/kg/day), pioglitazone (30 mg/kg/day), and combination (vidalgliptin 10 mg/kg/day and pioglitazone 30 mg/kg/day), (B) Change in body weight during the treatment with control, vildagliptin, pioglitazone, and combination. All data represented as Mean ± SD. * *p* < 0.05, ** *p* < 0.01, *** *p* < 0.001 (combination group) and ^#^ *p* < 0.05, ^##^ *p* < 0.01, ^###^ *p* < 0.001 (pioglitazone group) compared with control group.

Rats were administered control (vehicle), vildagliptin (10 mg/kg), pioglitazone (30 mg/kg) and combination treatment (vildagliptin 10 mg/kg and pioglitazone 30 mg/kg) for 5 weeks. The daily glucose levels of rats that were fasted for 4 h were found to be similar in vildagliptin group with control group. From week 2 to week 5, blood glucose levels in pioglitazone and combination group were found to be significantly lower than in the other groups (Fig 1A). Administration of both pioglitazone and combination caused an increase in the body weight during the treatment; however, vildagliptin treatment alone did not (Fig 1B).

### Glucose profile and insulin secretion after 5 weeks of treatment in ZDF rats

**Fig 2:**
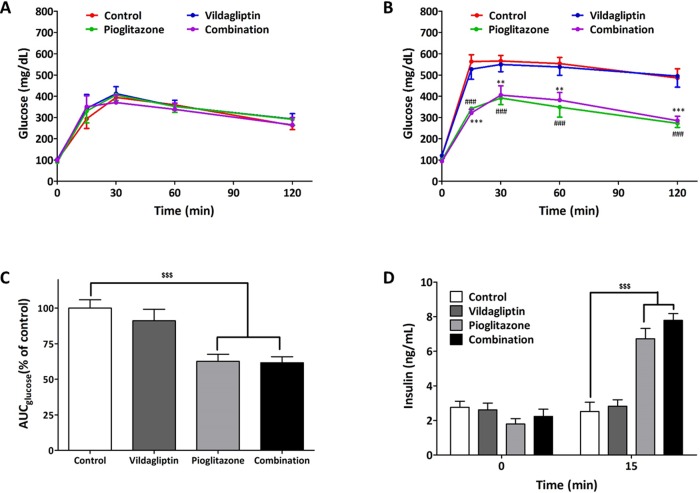
Glucose profile and insulin secretion after 5-week treatment in ZDF rats. (A) Glucose profile in IPGTT (glucose 2g/kg) before treatment (B) Glucose profile in IPGTT (glucose 2 g/kg) after treatment with control, vildagliptin (10 mg/kg/day), pioglitazone (30 mg/kg/day), and combination (vidalgliptin 10 mg/kg/day and pioglitazone 30 mg/kg/day). (C) AUC of glucose during the IPGTT after treatment (D) serum insulin level at 0 and 15 min in IPGTT after treatment. All data represented as Mean ± SD. **p < 0.01 and ***p < 0.001 (combination group) and ^###^ *p* < 0.001 (pioglitazone group) compared with control group. ^$$^*p* < 0.01, ^$$$^*p* < 0.001 compared with control group.

We performed IPGTT at the beginning and end of treatment. Generally ZDF rats got postprandial hyperglycemia from 12-week-old and developed diabetes unlike Zucker lean rats having glucose levels below 200 mg/dL during IPGTT. In all groups of animals, glucose levels at 120 min glucose load were near 300 mg/dL in IPGTT (Fig 2A) and we thought that diabetes was developed in animals and started the treatment. The glucose profiles of control and vildagliptin treatment group worsened progressively after 5 weeks of treatment. However, treatment with pioglitazone and combination of pioglitazone and vildagliptin was found to protect the progression of diabetes (Fig 2B). The AUC of glucose showed similar findings (Fig 2C). There was no significant difference in the serum insulin levels across all groups at 0 min, however, serum insulin levels at 15 min increased significantly in the pioglitazone and combination group as compared to that in the control group (Fig 2D).

### Bone mineral density and bone morphology after 5 weeks of treatment in ZDF rats

**Fig 3:**
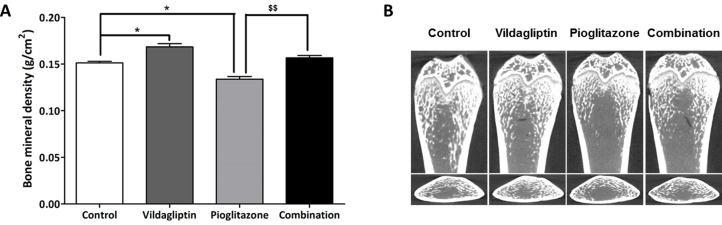
Bone mineral density and bone morphology after 5 weeks of treatment in ZDF rats. (A) Whole body bone mineral density (BMD) after treatments with control, vildagliptin (10mg/kg/day), pioglitazone (30 mg/kg/day), and combination (vidalgliptin 10 mg/kg/day and pioglitazone 30 mg/kg/day), (B) Micro-Computed Tomography (micro-CT) of femur after treatments with control, vildagliptin, pioglitazone, and combination. All data represented as Mean ± SD. *p < 0.05 compared with control group. ^$$^*p* < 0.01 compared with pioglitazone group.

We performed whole body DEXA analysis and micro-Computed Tomography (micro-CT) bone analysis after 5 weeks of treatment. The results revealed that the BMD increased significantly in the vildagliptin group and decreased in the pioglitazone group as compared to that in the control group. In addition, treatment with vildagliptin and pioglitazone (i.e. combination group) was found to protect bone loss due to treatment with pioglitazone alone (Fig 3A). Results of micro-CT bone analysis were in accordance with the results of BMD (Fig 3B). The ratio of trabecular bone volume over the total tissue volume (BV/TV) was significantly higher and the trabecular space (Tb.Sp) was significantly lower in vildagliptin group than in the control group. In contrast, pioglitazone treatment decreased BV/TV, trabecular thickness (Tb.Th), and trabecular number (Tb.N), and increased Tb.Sp significantly. Additional vildagliptin treatment (i.e. combination treatment) ameliorated pioglitazone-induced bone loss (Table 1).

**Table 1:** Bone histomorphometric results in ZDF rats.

	Control	Vildagliptin	Pioglitazone	Combination
Trabecular bone volume (BV/TV) (%)	72.69 ± 4.6	79.68 ± 2.0**	59.00 ± 2.5***	65.27 ± 2.6 ^$$^
Trabecular thickness (μm) (Tb.Th)	0.52 ± 0.03	0.61 ± 0.02	0.38 ± 0.01***	0.40 ± 0.01^$$$^
Trabecular number (mm^-1^) (Tb.N)	0.83 ± 0.02	0.88 ± 0.03	0.65 ± 0.01**	0.84 ± 0.04^$$$^
Trabecular space (μm) (Tb.Sp)	0.69 ± 0.03	0.54 ± 0.04*	1.15 ± 0.02***	0.79 ± 0.05

* *p* < 0.05

** *p* < 0.01

*** *p* < 0.001 versus control group.

^$$^ *p* < 0.01

^$$$^ *p* < 0.001 versus pioglitazone group.

### Serum active GLP-1, bone turnover markers, and sclerostin after 5 weeks of treatment in ZDF rats

**Fig 4:**
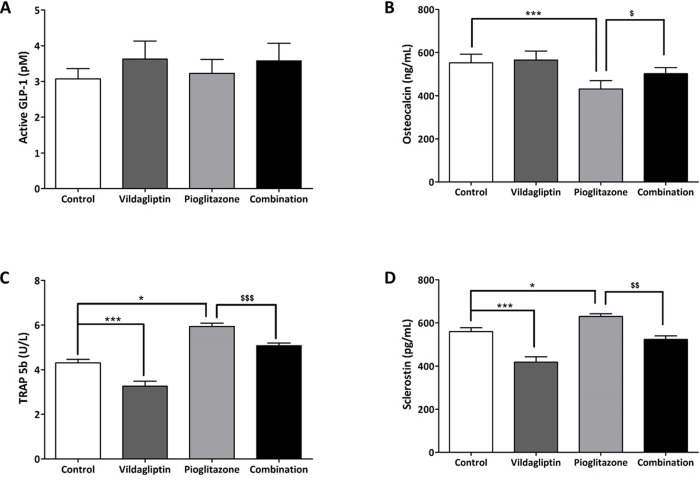
Active GLP-1, bone turnover markers (osteocalcin and TRAP 5b), and sclerostin levels in ZDF rats. (A) serum active GLP-1 levels, (B) serum osteocalcin levels, (C) serum TRAP5b levels, (D) serum sclerostin levels. Blood sampling was done after 5 weeks of treatment with control, vildagliptin (10 mg/kg/day), pioglitazone (30 mg/kg/day) and combination (vildagliptin 10 mg/kg/day and pioglitazone 30 mg/kg/day). And all data represented as Mean ± SD. *p < 0.05 ***, *p* < 0.001 compared with control group. ^$^ *p* < 0.005, ^$$^ *p* < 0.01, ^$$$^ *p* < 0.0001 versus pioglitazone group.

Vildagliptin treatment caused an insignificant increase in serum active GLP-1 levels (Fig 4A). We measured serum osteocalcin as a bone formation marker and serum TRAP-5b as a bone resorption marker. Osteocalcin levels were found to decrease significantly in the pioglitazone group and were restored in the combination group. TRAP 5b level increased significantly in the pioglitazone treated group, and decreased in the combination group. There was no change in osteocalcin levels, but a significant decrease in the TRAP 5b levels was observed in the vildagliptin group (Fig 4B and 4C). To evaluate the factor affecting bone formation and resorption, we measured the levels of sclerostin in the serum. Sclerostin levels were found to increase significantly in the pioglitazone group, and decrease to control values in the combination group. Additionally, there was a significant decrease in sclerostin levels in the vildagliptin group as compared to that in the control group (Fig 4D).

## Discussion

Diabetes mellitus is one of the most common chronic diseases with increasing prevalence worldwide. This rapid increase is related to population growth, aging, urbanization, and increasing prevalence of obesity and physical inactivity [12,13]. T2DM is the most common type of diabetes and is associated with insulin resistance. Clinical evidences show that T2DM patients have an increased risk of fractures regardless of normal or even high BMD. This may be associated to poor bone quality related to prolonged hyperglycemia and increased risks of falling, due to complications such as neuropathy, vascular disease, and impaired vision [3]. Additionally, it is also associated with the use of antidiabetic drugs such as TZDs.

TZDs are used as glucose-lowering agents that exhibit a beneficial effect on insulin sensitivity and cause an improvement in lipid metabolism [14,15]. However, the prolonged use of TZDs is associated with bone loss. Several preclinical studies have demonstrated that TZDs decrease BMD and trabecular bone volume and play a role in increasing bone resorption and decreasing bone formation by suppressing osteoblast differentiation in animal models [16–18]. Clinical studies also demonstrated that use of TZDs causes bone loss and increases the risk of fractures in humans, specifically in postmenopausal women with T2DM [19–21]. In line with the findings from earlier studies, the results of our study showed that pioglitazone decreased the BMD and trabecular bone volume in a diabetic animal model, and this decrease correlated with an increase in bone resorption and decreased bone formation.

In recent times, incretin-based therapy has been prescribed in the treatment of diabetes for T2DM patients worldwide. Major incretin hormones secreted from gut are GLP-1 and Glucose-dependent insulinotropic polypeptide (GIP). As these incretin hormones are rapidly degraded by DPP4 enzyme, DPP4 inhibition results in increased blood concentration of GLP-1 and GIP. In incretin-based therapy, GLP-1 analogs and DPP4i are used. GLP-1 is released from the L-cells in response to nutrient intake, stimulates glucose-dependent insulin release, and inhibits glucose-dependent glucagon release. In addition, GLP-1 reduces appetite, inhibits gastric emptying [22], and possibly exhibits positive effects on the bone unlike TZDs [9,23,24]. In a previous study, we have already shown the possibility that exendin-4 might increase the BMD by decreasing the expression of SOST/sclerostin in osteocytes in T2DM. Exendin-4 was found to reduce the serum levels of sclerostin, increase the levels of osteocalcin, and increase the femoral BMD in a type 2 diabetic animal model [10]. Recently some studies reported that another incretin, GIP also may have a positive effect on bone strength and quality [25,26].

DPP4 inhibitors are more widely used than GLP-1 RA since they can be administered orally while GLP-1 RA has to be administered parenterally. Recently, it was reported that sitagliptin treatment was found to diminish bone loss and increase mechanical bone strength in male streptozotocin-induced diabetic rats without any effects on glucose levels [11]. However, saxagliptin was reported to alter the long-bone microarchitecture and reduce the osteogenic potential of bone marrow stem cells [27]. While the effect of DPP4i on bone has not been confirmed thus far, it is expected to have a positive effect on the bone like GLP-1 RA.

Some studies have shown that DPP4i and TZD combination therapy has several advantages. Vildagliptin and pioglitazone combination therapy in patients with T2DM provided better glycemic control and reduction in the risk of hypoglycemia than either monotherapy [28]. Alogliptin/pioglitazone combination therapy improved β-cell function in patients with recent-onset T2DM [29]. Clinically, DPP4i and TZD combination therapy is commonly used for diabetes management. Therefore, we hypothesized that addition of a DPP4i in therapy might help in protection of TZD-induced bone loss.

Our study provides evidence for the first time that DPP4i can protect TZD-induced bone loss. In our study, we evaluated both, the action of vildagliptin on bone metabolism and the protective effects of vildagliptin on pioglitazone-induced bone loss in Zucker diabetic male rats. Vildagliptin and combination treatment increased the BMD and bone quality. To understand the mechanism of these bone histometric changes, we evaluated the levels of active GLP-1 and bone metabolic biomarkers. The active GLP-1 levels tended to increase in the vildagliptin and combination treatment groups compared to that in the other two groups though there was no significant difference. We thought that it might be related to use of minimum effective dose of vildagliptin (once a day). The level of the bone formation marker, osteocalcin, was found to decrease and that of the bone resorption marker, TRAP-5b, was found to increase in the pioglitazone group. The level of osteocalcin was found to increase and that of TRA-5b was found to decrease by additional treatment of vildagliptin in combination group. Sclerostin is expressed in osteocytes. Activation of sclerostin induces osteoblastic apoptosis and decreases bone formation. Contrary to this, inactivation of sclerostin induces osteoblast activity and increases bone formation [30,31]. TZDs were reported to induce osteocyte apoptosis and increase sclerostin expression [32]. We confirmed that the levels of sclerostin decreased significantly in the vildagliptin group, increased in the pioglitazone group, and decreased again in the combination group. It is suggested that the downregulation of sclerostin expression in osteocytes might be related to positive bone effects. We suggest that there are some possibilities of these bone effects; direct bone effect of DPP4i irrelevant to GLP-1, indirect bone effect via incretin, or both. In our previous study, we reported that exendin-4 treatment increased the levels of osteocalcin in the serum and decreased the levels of sclerostin without causing any change in the levels of TRAP-5b [10]. But there are other possibilities. DPP4 is also known Cluster of differentiation 26 (CD 26). Recent study showed that inhibition of CD 26 signaling inhibited human osteoclast development and humanized anti-CD26 monoclonal antibody may have therapeutic potential for the treatment of osteolytic lesions [33]. Recently some studies reported that DPP4i had anti-inflammatory effects and it also becomes the another possibility [34,35]. And increased levels of GIP might be able to affect the result though we did not measure [25,26]. Further studies are warranted to explore the mechanism.

In our study, we evaluated the effects of vildagliptin on the bone, and its protective role against TZD-induced bone loss in ZDF rats. Our results are in agreement with our expectations; however, the present study has certain limitations. We measured the concentration of sclerostin in the plasma by ELISA; however, we did not directly measure the levels in the bone using western blot or immunohistochemistry. Thought there have been some previous reports of protective effects of GIP on the bone [25,26] the present study focused on the effect of GLP-1 and measured the levels of active GLP-1 without measuring the levels of GIP. Additionally, we did not measure the levels of calcitonin, since the calcitonin levels were not increased by exendin-4 in our previous study [10].

In conclusion, vildagliptin, a DPP4i, increased bone mass in a T2DM animal model and a combination treatment of vildagliptin and pioglitazone ameliorated the bone loss caused by pioglitazone. Thus, clinically, treatment using a combination of DPP4i and TZD is expected to minimize bone loss and fracture risk in T2DM patients treated by TZDs.
